# NOD2 inhibits the proliferation of esophageal adenocarcinoma cells through autophagy

**DOI:** 10.1007/s00432-022-04354-x

**Published:** 2022-10-31

**Authors:** Xiaozhi Li, Suo Liu, Longyu Jin, Yuchao Ma, Tao Liu

**Affiliations:** 1 Emergency Department, The Third XiangYa Hospital, Central South University, Changsha, 410013 Hunan China; 2 Cardiothoracic Surgery, The First Affiliated Hospital, Hengyang Medical School, University of South China, Hengyang, 421001 Hunan China; 3 Cardiothoracic Surgery, The Third XiangYa Hospital, Central South University, Changsha, 410013 Hunan China

**Keywords:** NOD2, Autophagy, Esophageal adenocarcinoma, Proliferation

## Abstract

**Aim:**

To study the regulatory mechanism of NOD2 in the inhibition of esophageal adenocarcinoma cell proliferation.

**Methods:**

Cell experiments: after confirming the decrease in NOD2 expression in esophageal adenocarcinoma, we overexpressed NOD2 in esophageal adenocarcinoma cells via lentivirus, compared and verified the changes in esophageal adenocarcinoma cell proliferation before and after NOD2 overexpression, and compared the overexpression group with the control group by mRNA sequencing to identify pathways that may affect cell proliferation. Then, the autophagy level of multiple groups were assessed, and the results were verified by rescue experiments. In vivo experiments: we administered esophageal adenocarcinoma cells to nude mice to form tumors under their skin and then injected the tumors with NOD2 overexpression lentivirus and negative control lentivirus. After a period of time, the growth curve of the tumor was generated, and the tumor was removed to generate sections. Ki67 was labeled with immunohistochemistry to verify cell proliferation, and the protein was extracted from the tissue to detect the molecular indices of the corresponding pathway.

**Results:**

Upregulation of NOD2 expression inhibited the proliferation of esophageal adenocarcinoma cells. Upregulation of NOD2 expression increased the autophagy level of esophageal adenocarcinoma cells via ATG16L1. After ATG16L1 was inhibited, NOD2 had no significant effect on autophagy and proliferation of esophageal adenocarcinoma cells. Enhanced autophagy in esophageal adenocarcinoma cell lines inhibited cell proliferation. In vivo, the upregulation of NOD2 expression improved the autophagy level of tumor tissue and inhibited cells proliferation.

**Conclusion:**

NOD2 can activate autophagy in esophageal adenocarcinoma cells through the ATG16L1 pathway and inhibit cell proliferation.

## Introduction

Esophageal adenocarcinoma (EA), a kind of cancer originating from the esophageal epithelium, has different incidence rates in different regions. Adenocarcinoma is the main type of EA in developed countries (Coleman et al. 2018), and China also has a high incidence of this disease. At present, the treatment of EA is mainly surgical treatment supplemented by chemotherapy, targeted therapy and immunotherapy (Lagergren et al. 2017), but due to the difficulty of EA detection in the early stage, most diagnoses are in the late stage, which usually leads to a poor prognosis (Watanabe et al. 2020; Lyons and Ku 2017), and the surgical window is often missed. Neoadjuvant chemotherapy has risen and prevailed in many European countries in recent years (Vitz et al. 2018; Burt et al. 2017), it has been found that could improve the survival rate of patients with esophageal adenocarcinoma, but this scheme has not completely broken through the limitations of radiotherapy and chemotherapy, and will still be limited by various factors (Corsini et al. 2021). At the same time, targeting classic therapeutic targets, such as EGFR, MTOR, and MET, has been proven to be ineffective in patients with poor prognosis of esophageal cancer (Lyons and Ku 2017). Although PD1, VEGF and HER2 inhibitors have been proven to improve the survival rate of advanced EA and some of them have been approved as first-line and second-line treatments, the survival rate of EA is still very low (Barsouk et al. 2019). Therefore, currently, we lack an effective, broad-spectrum targeted drug for esophageal cancer, and we hope to establish a new treatment model for EA to improve its survival rate.

Autophagy is a process by which cells purify themselves (Kroemer et al. 2010), which is used to maintain the energy balance of cells and remove waste products. This process is conducive to the survival and growth of cells under normal conditions. However, excessive autophagy can also lead the cell to undergo apoptosis. Autophagy acts as a “double-edged sword” in tumor cells as it does in normal cells (Antunes et al. 2018; White et al. 2015). How to make this “double-edged sword” play a positive role in the treatment of tumors through external intervention, such as drug stimulation or gene editing, has always been a hot research topic among scientists(Hong et al. 2020).

Nucleotide binding oligomerization domain containing 2 (NOD2), which belongs to the NOD receptor family inside the cytoplasm, is an innate immune receptor for bacteriogenic components activated by muramyl dipeptide (MDP). MDP can promote the aggregation of the autophagy-related gene ATG16L1 to the site where bacteria enter the cells, activate the autophagy of dendritic cells and induce their apoptosis (Caruso et al. 2014; Al Nabhani et al. 2017). Although NOD2 is well known for its role in inflammatory responses, its role in cancer has been widely debated, with some suggesting that NOD2’s inflammatory response promotes cancer (Angeletti et al. 2009; Hnatyszyn et al. 2019) and others suggesting that it has anticancer effects based on data analysis of a number of genetic mutations (Li et al. 2015; Zhang et al. 2020; Huszno et al. 2020). Therefore, this study mainly explored the possible applications of NOD2’s regulation of autophagy in the treatment of EA, and the results may identify a new therapeutic approach for esophageal adenocarcinoma.

## Materials and methods

### Experimental materials

Het-1A, OE33, SEG-1 and BIC-1 cell lines were obtained from ATCC (Gaithersburg, MD, USA). NOD2 overexpression lentivirus, Negative control lentivirus (NOD2-OV), ATG16L1-shRNA lentivirus, Negative control lentivirus (sh-ATG16L1), RFP-GFP-LC3 lentivirus (GeneChem, Shanghai, CN), an electron microscope dyeing kit (Ted Pella, Inc.), rapamycin (Selleck, Texas, USA), an EDU experimental kit, CCK-8 reagent, a co-IP kit, an immunohistochemical kit (Biosharp, Anhui, CN), mRNA primer (Tsingke, Beijing, CN), RNA Extraction Kit (Accurate, Hunan, CN), and a reverse transcription kit (Toyobo, Japan) were obtained, and genome sequencing was used (GeneChem, Shanghai, CN).

### Experimental methods

#### Cell culture

The human Het-1A, OE33, SEG-1 and BIC-1 cell lines were cultured in Dulbecco’s modified Eagle’s medium (DMEM, Gibco) with 10% fetal bovine serum (FBS, Gibco), 1% penicillin and streptomycin (100 U/mL, Gibco) in a humidified incubator with 5% CO_2_ at 37 °C.

#### Tissue collection and ethics statement

Twelve primary esophageal adenocarcinoma patients undergoing tumor resection were recruited at the Third Xiangya Hospital of Central South University (Changsha, China) from September 2018 to December 2020. Appropriate ethical approval was obtained from the Third Xiangya Hospital Ethics Committee, and written informed consent was obtained from all patients. Fresh esophageal adenocarcinoma tumor tissues and their adjacent nonmalignant lung tissues were sampled and stored at − 80 °C.

#### EDU assay

Cell proliferation was measured by a 5-ethynyl-29-deoxyuridine (EDU) assay using an EDU assay kit (Biosharp, Anhui, CN) according to the manufacturer’s instructions. Briefly, OE33 cells were cultured at a density of 1*10^4 cells/well in 100 μL of medium in 96-well microplates (Biomedical Engineering) for 24 h. Then, the OE33 cells were grouped and treated according to the experimental needs for 24 h. The cells were then exposed to 50 mM EDU for an additional 3 h at 37 °C. The cells were then fixed with 4% formaldehyde for 15 min at room temperature and treated with 0.5% Triton X-100 for 20 min at room temperature for permeabilization. After 3 washes with PBS, the cells were treated with 100 μL of 16 Apollo reaction cocktail for 30 min. Subsequently, the DNA contents of each well of cells were stained with 100 μL of Hoechst 33,342 (5 mg/mL) for 30 min and visualized under a fluorescence microscope (Olympus IX-73, Japan).

#### CCK-8 assays

Cell viability was analyzed by Cell Counting Kit-8 (CCK-8, Beyotime, Shanghai, China) according to the manufacturer’s protocols. Cells were seeded and cultured at a density of 2*10^3/well in 100 μL of medium in 96-well microplates (Biomedical Engineering). Then, the cells were grouped and treated according to the experimental needs. After treatment for 0, 24, 48, and 72 h, 10 μL of CCK-8 reagent was added to each well and then cultured for 2 h. All experiments were performed in triplicate. The absorbance was analyzed at 450 nm using a multiclan spectrum (Envision Xcite) using wells without cells as blanks. The proliferation of cells was determined by the absorbance.

#### Plate cloning assay

Cell suspensions were prepared, and their concentrations were measured and diluted to 500 cells/mL with DMEM. Two wells from a 6-well plate were used for each cell line (Biomedical Engineering), and 2 mL of cell suspension (1000 cells) was inoculated in each well, incubated at 37 °C for 12 days, gently washed with PBS, and fixed with 500 μL of paraformaldehyde for 20 min. Then, the cells were stained with crystal violet dye for 30 min, washed with PBS, dried and photographed.

#### Transfection assay of RFP-GFP-LC3

EA cells were allowed to grow in sheets in six-well plates, with approximately 10,000 cells per well. The cells were infected with RFP-GFP-LC3 lentivirus, incubated at 37 ℃ for 12 h, washed to remove the virus solution, cultured for 12 h, and observed, and images were collected under a fluorescence microscope (Olympus, IX-73).

#### Real-time PCR (RT-PCR) detection

An Accurate Biotechnology kit was used, and 300 μL of lysate was added to each well of a 6-well plate. After cell lysis, RNA was extracted according to the instructions. A Nanodrop was used to detect the RNA concentration. After dilution with deionized water, RNA was stored at − 80 °C. One microgram of RNA was used, and it was treated at 65 °C for 5 min. The RNA was used to generate cDNA using a Toyobo kit, diluted with deionized water and stored in a refrigerator at − 20 °C. The amplification conditions of RT-PCR were as follows: predenaturation at 95 °C for 2 min and denaturation at 95 °C for 10 s, annealing at 58 °C for 10 s, and extension at 68 °C for 30 s, 50 cycles. The primer sequences are shown in Table 1 (Tsingke, Beijing, CN). With GAPDH as the internal reference gene, the relative mRNA expression of each target gene was calculated with 2^− ΔΔCT^.

**Table 1:** Primer sequences

Primer names	Primer sequences
NOD2-F	5′-CTCAGCTTCCCAAGGTCTGG-3′
NOD2-R	5′-AGGTAGAACGCGGCAAAGAA-3′
GAPDH-F	5′-GTGACGTGGACATCCGCAAAG-3′
GAPDH-R	5′-GGAGAATGGACAGCGAGGC -3′

#### Western blot detection

Cell or tissue samples were separately harvested and lysed in RIPA buffer (CWbio, Beijing, China) containing 0.1 mg/mL PMSF (Keygen, Nanjing, China), protease inhibitor, and Phospho-stop (Roche, Mannheim, Germany). Protein aliquots (30 lg) were separated by 10% SDS-PAGE and transferred to 0.45-lm PVDF membranes (Millipore, Billerica, MA, USA). The blots were blocked for 1 h at room temperature and incubated separately with primary antibodies (diluted 1:1000) against ATG16L1, P-ATG16L1, LC3I/II, SQSTM1/P62 (Abclonal, Massachusetts, USA) (rabbit anti human), NOD2 (Novus, Missouri, USA) (mouse anti human), and GAPDH (Proteintech, Wuhan, China) (rabbit anti human) overnight at 4 °C. The PVDF membranes were washed with TBS–Tween 20 and then incubated separately with the appropriate HRP-conjugated secondary antibodies (diluted 1:5000) (CST, Massachusetts, USA) (goat anti-mouse, goat anti-rabbit) for 1 h at room temperature. After rinsing, the signal on the PVDF membrane was detected by the enhanced chemiluminescence method. The relative protein expression is presented as the ratio of target protein band intensity to GAPDH band intensity using ImageJ software (NIH, Bethesda, MD, USA).

#### Co-IP assay

After the cells were lysed with RIPA lysis buffer to extract protein, one part was separated into the input group, and NOD2/ATG16L1 antibody (In our research, the CO-IP experiment was carried out twice, the ATG16L1 was pulled down with NOD2 antibody for the first time, and then we pulled NOD2 protein down with ATG16L1 antibody.) was added to the remaining liquid (1 μg), which was slowly shaken at 4 ℃ and incubated overnight. Ten microliters of protein A agarose beads was washed with modified RIPA buffer lysis buffer 3 times and centrifuged at 3000 rpm for 3 min each time. The pretreated protein A agarose beads were added to the cell lysate that had been incubated with the antibody overnight and incubated at 4 ℃ for 4 h to couple the antibody with protein A agarose beads. After the immunoprecipitation reaction, the samples were centrifuged at 3000 rpm for 3 min at 4 °C, the supernatant was aspirated and discarded, the agarose beads were washed with modified RIPA buffer lysate 3 times, and 2 × SDS loading buffer (15 μL) and boiling water were added for 5 min. Binding proteins were determined by Western blot analysis.

#### In vivo tumor growth

Male Nc-Nu nude mice, 4–6 weeks old, were obtained from the Center for Medical Experiments of the Third Xiangya Hospital of Central South University. The research protocol was approved, and the mice were maintained according to the Institutional Guidelines of the Animal Ethics Committee of Central South University. Nude mice were randomized into two different groups (4 mice/group) and inoculated with OE33 cells (4 × 10^6 cells/100 μL) in the right axilla. When all tumors reached a mean diameter of 5 mm, the nude mice were treated with NOD2-overexpressing lentiviruses or lentiviral vector (5 × 10^7 TU/100 μL) by intratumor injection each week (all 3 times). The tumor length and width of each mouse were measured weekly by a digital caliper. The tumor volumes (V) were calculated using the following formula: V = length*(width^2). The relative tumor volume (RTV) was calculated by the following formula: RTV = Vw/V0, in which Vw represents the volume each week, and V0 is the initial tumor volume at the beginning of lentiviral treatment. All nude mice were killed 3 weeks after the first lentiviral treatment, and the tumor tissues were collected for analysis and photographed. Then, the protein was extracted from the tumor tissue, and the content of related indices was detected by Western blots.

#### Immunohistochemistry assay

After the tumor tissue was removed, it was fixed with paraformaldehyde. After fixation, the cells were placed in ethanol for gradient dehydration and then embedded in paraffin. After the wax was completely solidified, it was stored at 4 ℃. After the wax block was sliced with a slicer, it was placed into a 50 ℃ water bath to melt and stuck to the center of the slide. The slides were baked overnight in a 37 ℃ incubator and then stored in a 4 ℃ refrigerator. The slices were dewaxed, inactivated with 3% hydrogen peroxide for 5 min, and washed with distilled water 3 times. The slices were immersed in 0.01 M citrate buffer (pH 6.0) and heated in the microwave oven until boiling. Then, the power was turned off, the procedure was repeated twice at an interval of 5 min, and the samples were washed twice with PBS after cooling. Then, 5% BSA blocking solution was added dropwise for blocking at room temperature for 20 min to remove excess liquid. Then, 1:100 diluted Ki67 antibody (Proteintech, Wuhan, China, mouse anti-human) was added dropwise, and the cells were washed with PBS for 2 min overnight at 4 ℃ × three times. The cells were incubated with biotinylated goat anti-mouse IgG at 37 ℃ for 20 min and washed with PBS. SABC reagent was added dropwise at 37 ℃ for 20 min, and the cells were washed with PBS. A DAB color development kit was used to develop the color for 30 min. The sections were washed with distilled water, dehydrated, made transparent, sealed, observed and photographed with a microscope.

#### Electron microscopy

An agar solution of a 20 g/L was prepared with distilled water, heated to dissolve, and poured into a conical tube. After the EA cells were digested, they were added to a centrifuge tube and centrifuged at 1000 rpm/min for 5 min. The supernatant was discarded and resuspended in 5 mL of PBS. The suspended cells were added to an agar centrifuge tube and centrifuged at 2000 rpm/min for 15 min, and the supernatant was discarded. Then, 4% paraformaldehyde was added to fix for 15 min, the agar block was removed, and the cell mass was repaired with a knife, washed with PBS 3 times, fixed with 1% osmic acid (Ted Pella, Inc.) for 30 min, washed with distilled water 3 times, and dehydrated with 30%, 50%, 70%, 80%, 90%, 100% (I) and 100% (II) gradients for 2 min. The cell mass was soaked with resin (1:1 ethanol: resin for 60 min, 100% resin for 2 min) for 60 min and placed in a constant temperature oven (Liuyi, Beijing) at 60 ℃ for 2 h. The embedding agent was added in the capsule, and the label was placed. The agar block was moved to the center of the capsule, allowed to settle naturally to the bottom of the capsule, baked at 60 ℃ for 48 h, and sliced (Leica UC-7). The sections were stained with uranium acetate lead citrate and observed under an electron microscope (JEOL-TEM).

#### Data analysis

The results were analyzed using SPSS version 18 statistical software (IBM SPSS, Chicago, IL, USA). Normally distributed continuous variables were compared using ANOVA or the least significant difference *t* test, as appropriate. The statistical results are expressed as the mean ± SD of three independent experiments. A probability level of *P* < 0.05 was considered statistically significant.

## Results

### The expression of NOD2 was decreased in EA cells

**Fig. 1:**
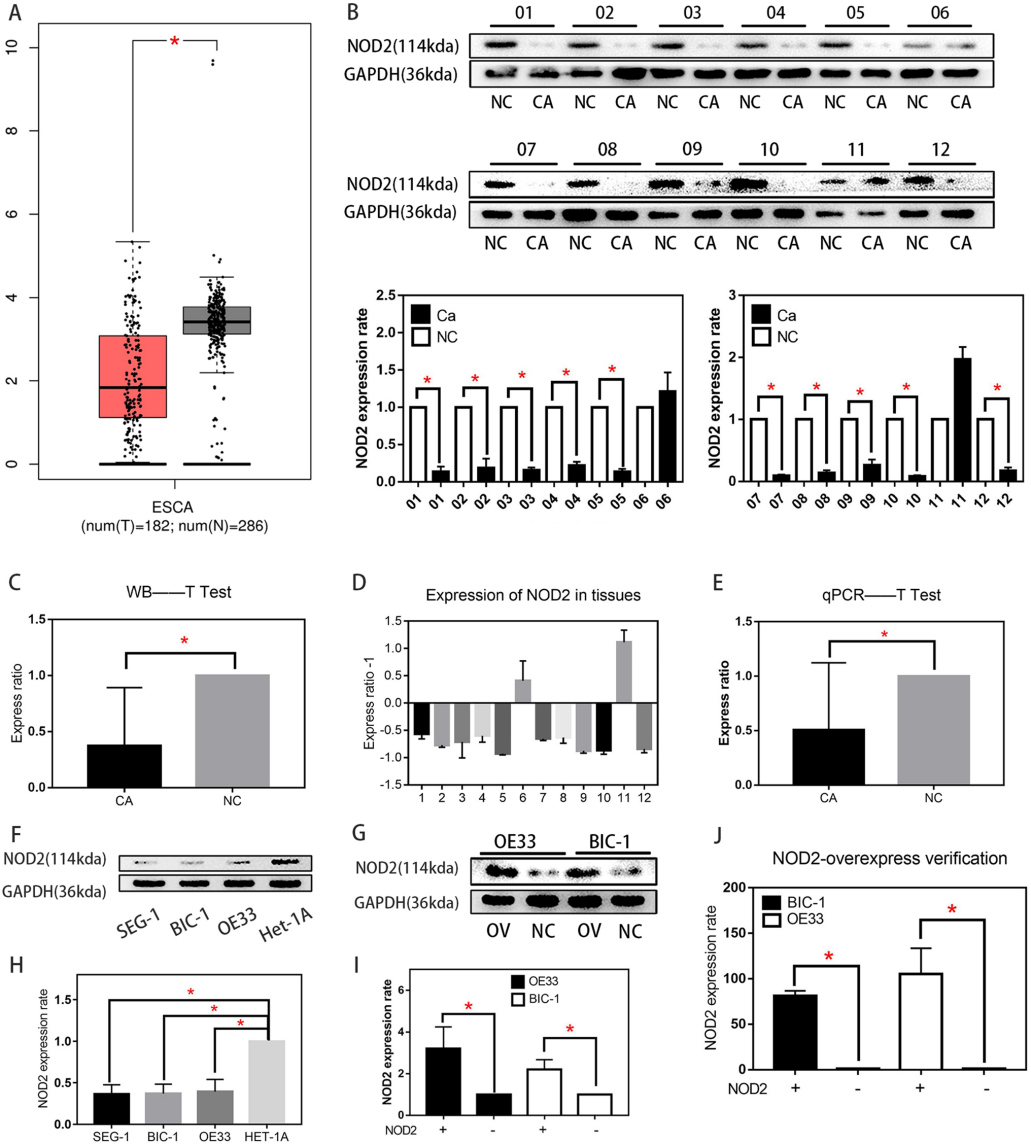
The expression of NOD2 was decreased in EA cells. **A** The Gene Expression Profiling Interactive Analysis (GEPIA) (http://gepia.cancer-pku.cn/) database showed that NOD2 levels were downregulated in the ESCA cell lines compared with the esophageal epithelial cell lines. **B** NOD2 protein expression in 12 pairs of tissue specimens was detected by Western blots. **C** Analyze the experimental results in (**B**) with *t* test. Statistical results are represented as the mean ± SD (*n* = 3, technical replicates, *P < 0.05). D, NOD2 mRNA expression in 12 pairs of tissue specimens was detected by RT-PCR. E, Analyze the experimental results in (**D**) with *t* test. Statistical results are represented as the mean ± SD (*n* = 3, technical replicates, *P < 0.05). **F** WB was used to detect the expression of NOD2 in three EA cell lines (SEG-1, BIC-1, OE33) and an esophageal epithelium cell line (Het-1A). **G** after stably transfected NOD2 overexpression cell lines were constructed, the difference in NOD2 protein expression in BIC-1 and OE33 cell lines was verified by WB. **H** Analyze the experimental results in (**F**) with *t* test. Statistical results are represented as the mean ± SD (*n* = 3, technical replicates, *P < 0.05). *I* statistical analysis of (**G**). Statistical results are represented as the mean ± SD (*n* = 3, technical replicates, *P < 0.05). **J** the difference in NOD2 m-RNA expression in BIC-1 and OE33 cell lines was verified by RT-qPCR. Statistical results are represented as the mean ± SD (*n* = 3, technical replicates, *P < 0.05)

The Gene Expression Profiling Interactive Analysis (GEPIA) (cancer-pku.cn) database showed that NOD2 expression was downregulated in the ESCA cell lines compared with the esophageal epithelium cell lines (Fig. 1A). These results are consistent with previous studies (Ma et al. 2020), which reported that NOD2 is a tumor suppressor gene. To verify the results, we collected tumor tissue samples and normal tissue samples from 12 patients with pathologically confirmed EA for Western blot assays (Fig. 1B, C) and RT-PCR assays (Fig. 1D, E). The results showed that the NOD2 protein content in the 10 groups of EA tissues was significantly higher than that in adjacent tissues. Western blot analysis was also used to verify the protein expression in OE33, BIC-1, SEG-1 and Het-1A cell lines. The results are shown in Fig. 1F, H. NOD2 protein expression in the BIC-1, OE33, and SEG-1 EA cell lines was also lower than that in the Het-1A esophageal epithelial cell line. To study the specific function of NOD2 in EA cells, we used the EA cell lines OE33 and BIC-1 for the analysis. First, NOD2 was overexpressed in these cell lines by lentivirus transfection, and then, stably transfected cell lines were screened by puromycin. Then, the transfection efficiency was detected by Western blotting and RT-PCR, and the results are shown in Fig. 1G, I, J.

### NOD2 inhibited the proliferation of EA cells

**Fig. 2:**
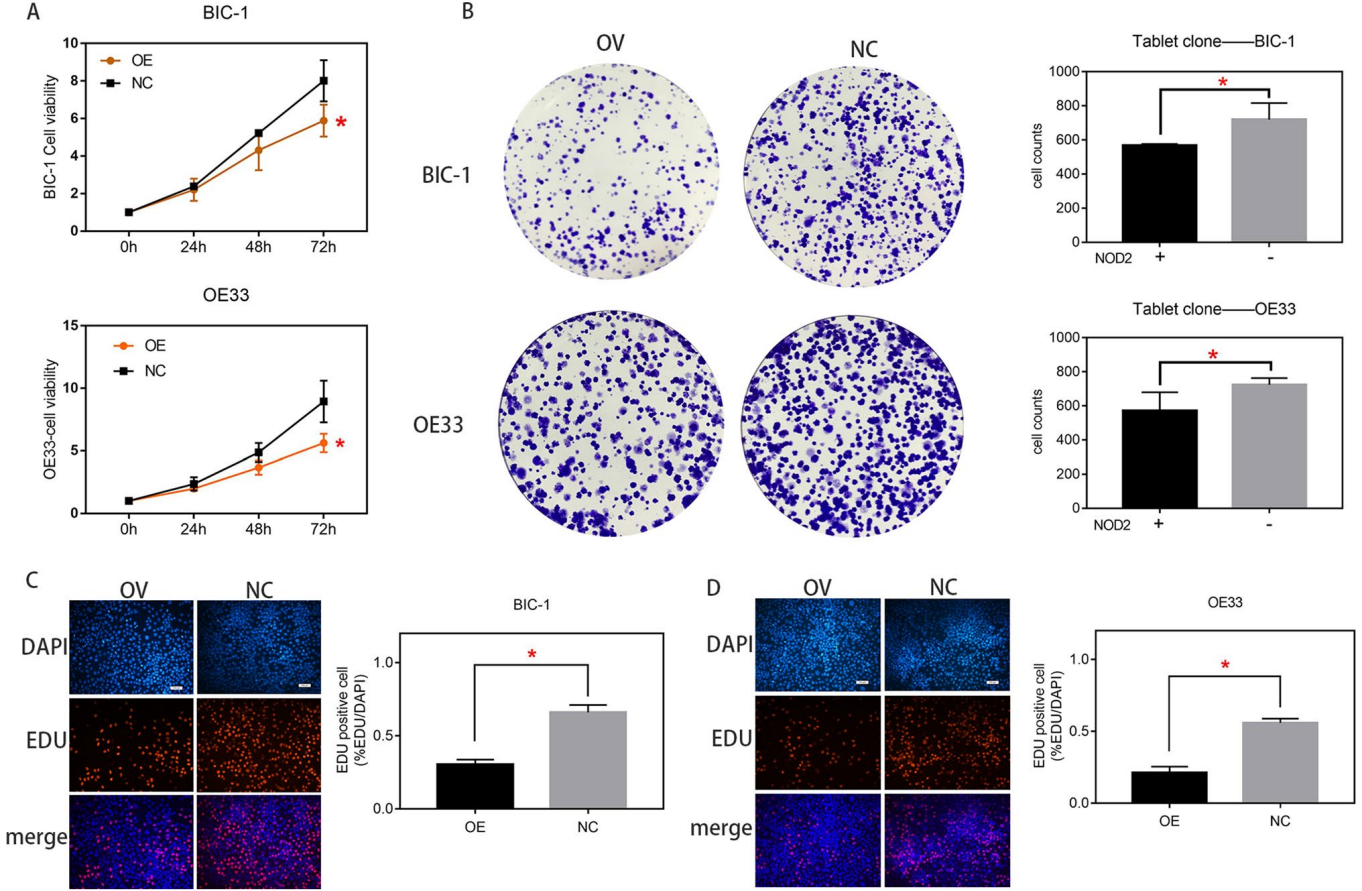
NOD2 inhibited the proliferation of EA cells. CCK-8 assays (**A**) and plate cloning assays (**B**) were carried out to measure the proliferation of the NOD2-overexpressing (OV) and negative virus treated control (NC) groups of OE33 and BIC-1 cells (*BIC-1-OV vs. BIC-1-NC, *OE33-OV vs. OE33-NC). **C**, **D** the proliferation of OV group and NC group of EA cells was detected by EDU assays (*BIC-1-OV vs. BIC-1-NC, *OE33-OV vs. OE33-NC). Statistical results are represented as the mean ± SD (*n* = 3, technical replicates, *P < 0.05)

To study the influence of NOD2 on EA, we performed CCK-8, plate cloning and EDU cell proliferation assays of OE33 and BIC-1 cell lines. Both CCK-8 and plate cloning assays showed that (Fig. 2A, B) the proliferation of EA cells was reduced due to the overexpression of NOD2, while the EDU assay also revealed that (Fig. 2C, D) the lentivirus overexpressing NOD2 could significantly inhibit the proliferation of EA cells.

### NOD2 overexpression inhibited the proliferation of EA cells

**Fig. 3:**
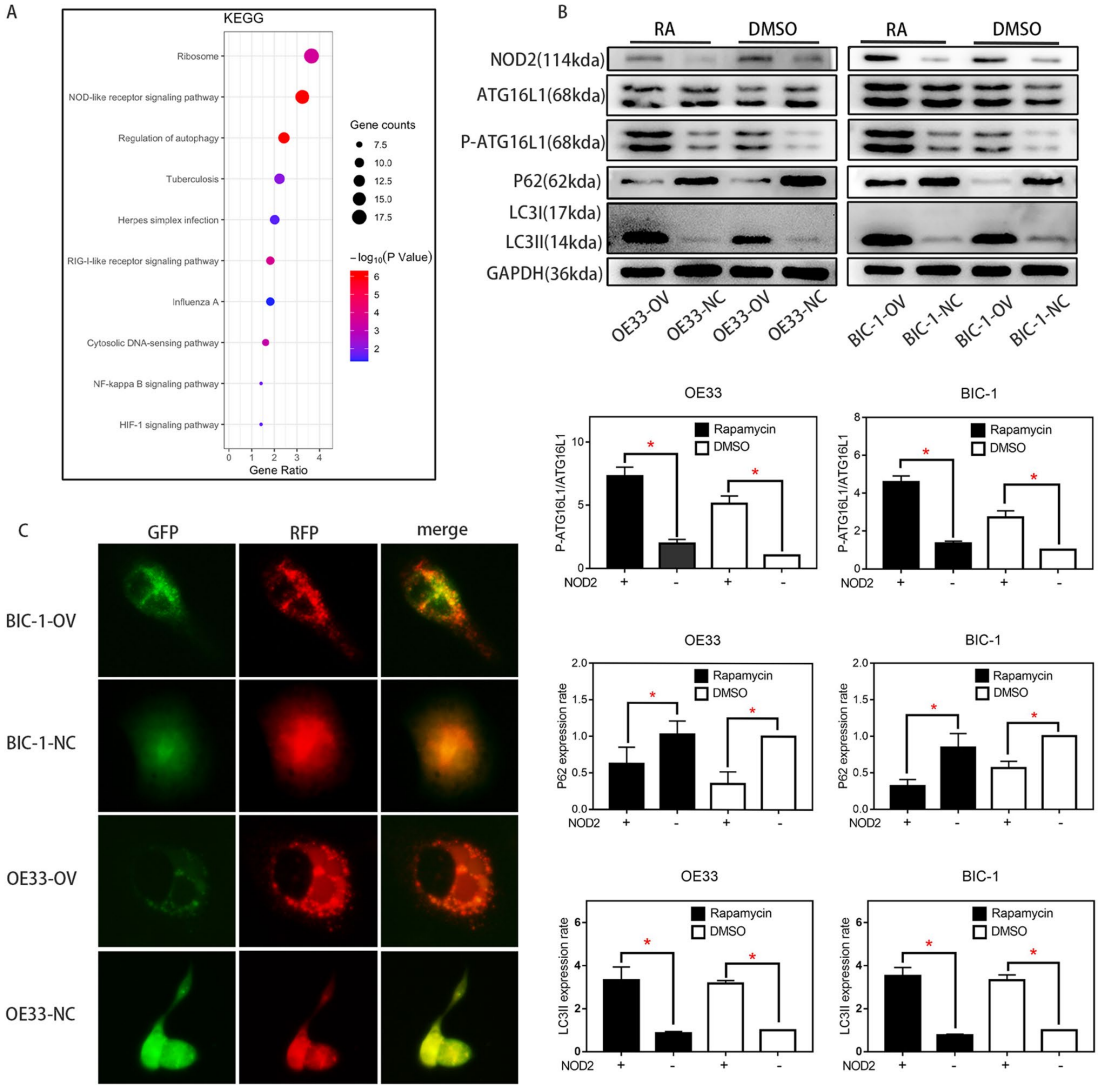
The effect of NOD2 overexpression on autophagy inhibited the proliferation of EA cells. **A** KEGG analysis of gene sequencing results showed that among the proteins affected by NOD2 overexpression in OE33 cells, a high proportion was related to “autophagy pathways”. **B** After rapamycin (100 nM/L) was used as an autophagy agonist, the expression levels of the autophagy-related proteins ATG16L1, P-ATG16L1, P62 and LC3I/II in the OV and NC groups of BIC-1 and OE33 cells were detected by Western blots. Statistical results are represented as the mean ± SD (*n* = 3, technical replicates, *P < 0.05). **C** after fluorescent labeling of LC3 protein with GFP and RFP, the autophagy levels of the OV group and NC group of BIC-1 and OE33 cells were detected. The results showed that the levels in the OV group were higher than those in the corresponding NC group

**Fig. 4:**
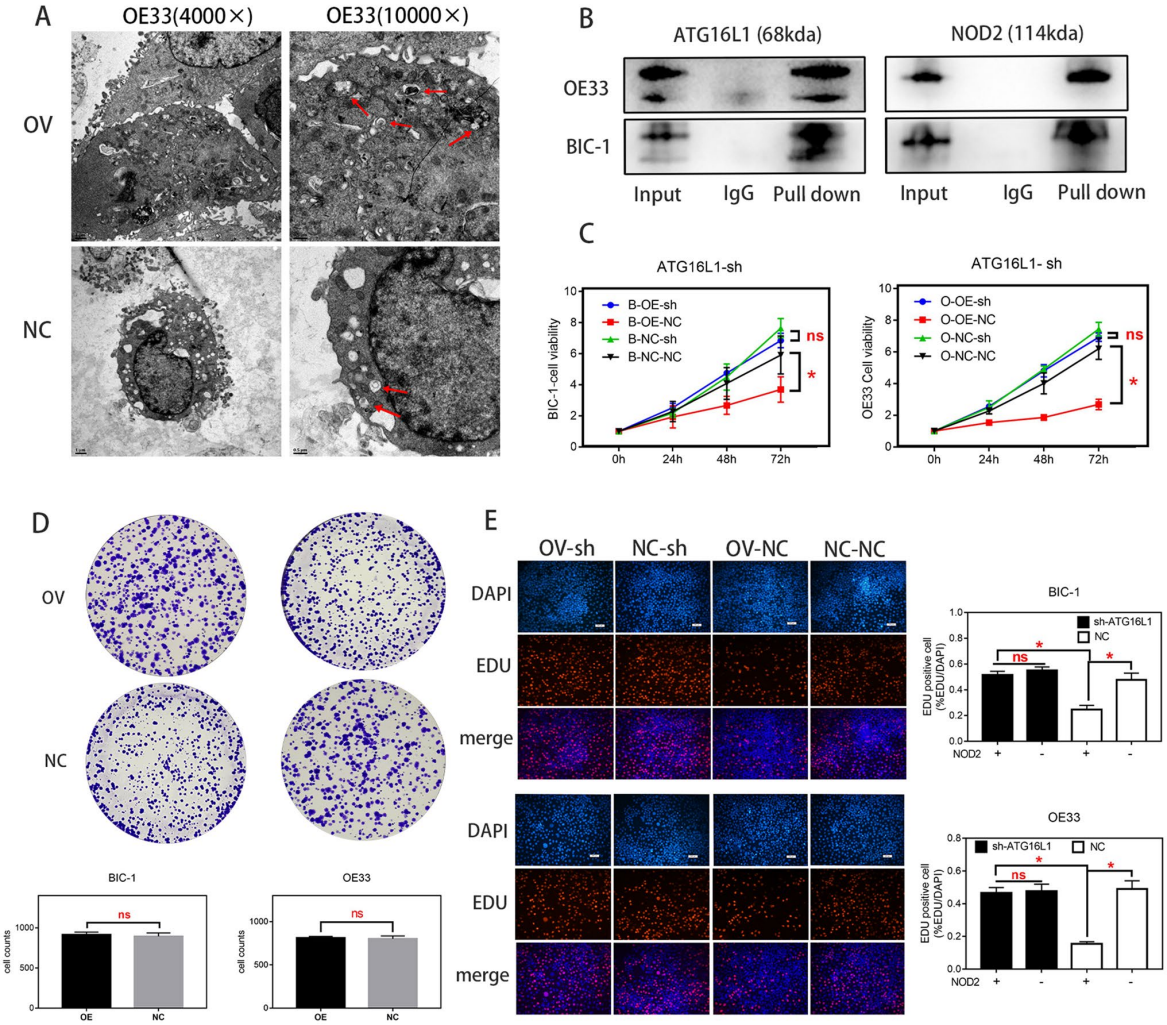
The effect of NOD2 overexpression on autophagy inhibited the proliferation of EA cells. **A** Electron microscopy showed that in BIC-1 and OE33 cells, the number of autophagic bodies in the OV group was higher than that in the NC group. **B** The interaction between NOD2 protein and P-ATG16L1 protein in BIC-1 and OE33 cells was detected by a co-IP assay. The results showed that there was a strong interaction between these proteins. **C**, **E** After ATG16L1-shRNA lentivirus (MOI = 50) was used as an ATG16L1 inhibitor, the proliferation of BIC-1 and OE33 cells in the A-sh group and the control group (Negative virus transfected with the same vector as ATG16L1-sh) was detected by CCK-8 (**C**) and EDU (**E**) assays. **D** The proliferation of BIC-1 and OE33 cells in the A-sh group and control group was detected by plate cloning assays. Statistical results are represented as the mean ± SD (*n* = 3, technical replicates, *P < 0.05, ns: *P* > 0.05)

**Fig. 5:**
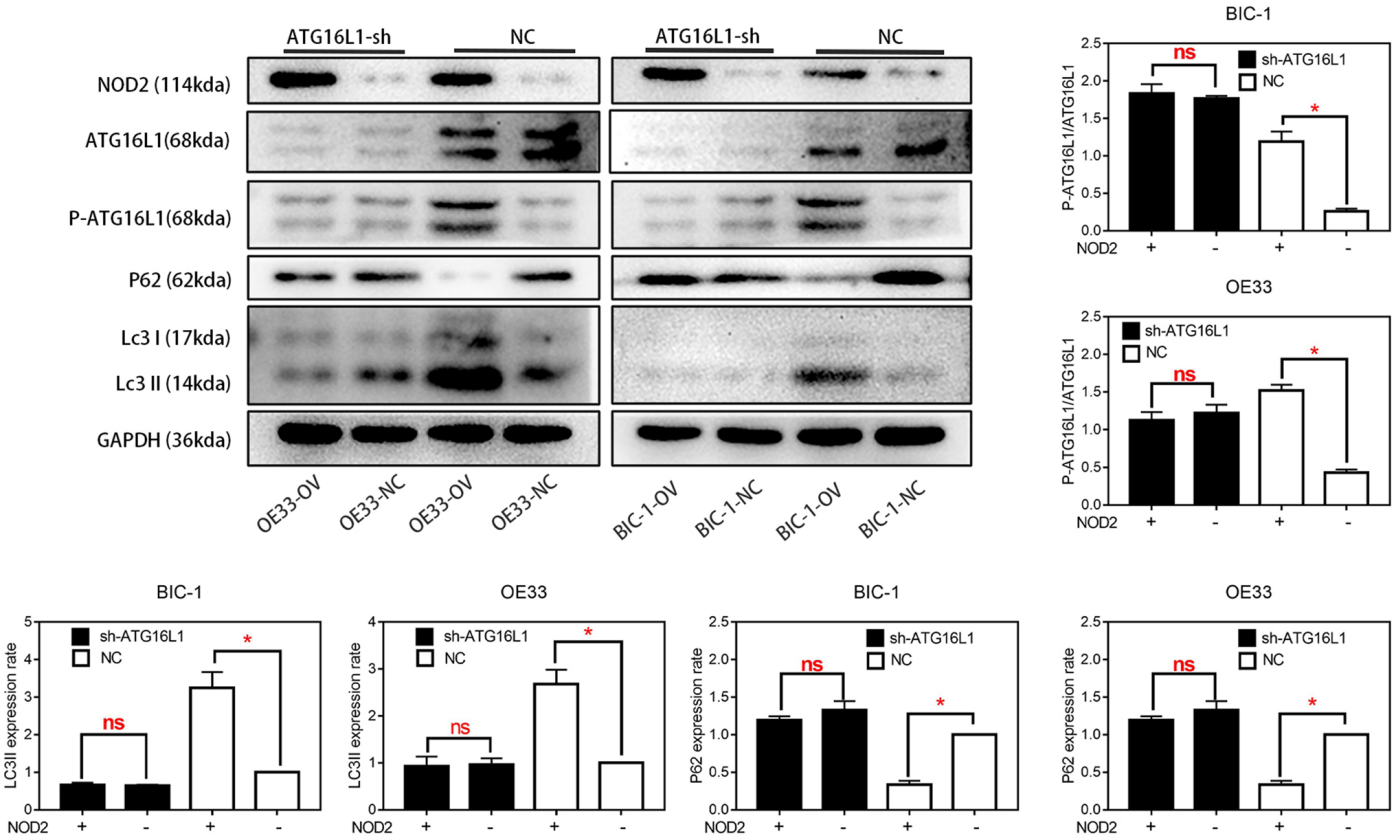
The effect of NOD2 overexpression on autophagy inhibited the proliferation of EA cells. When ATG16L1 was inhibited by sh-RNA lentivirus, and the control groups were treated by negative lentivirus same as ATG16L1-sh, the expression levels of ATG16L1, P-ATG16L1, P62 and LC3I/II in the OV and NC groups of BIC-1 and OE33 cells were detected by Western blots. Statistical results are represented as the mean ± SD (*n* = 3, technical replicates, *P < 0.05, ns: *P* > 0.05)

To confirm the regulation of NOD2 on autophagy in EA cells, we sequenced the mRNA of the OV group and NC group of OE33 cells and analyzed the pathways with KEGG analysis (http://metascape.org/gp/index.html#/main/step1), which could show us that which was the most affected pathway in OE33 cells after NOD2 was overexpressed. As shown in Fig. 3A, through the comparison of the OV group and the NC group, we found that in OE33 cells, the “autophagy pathway” was strongly regulated by NOD2. This finding was preliminarily verified by Western blot experiments. As shown in Fig. 3B, in the EA cell lines, the upregulation of NOD2 expression significantly increased the phosphorylation level of ATG16L1, which then increased the autophagy level of the cells, as confirmed by the decrease in P62 and the enhancement of LC3II. Upregulated NOD2 expression increased the phosphorylation of ATG16L1 more significantly after autophagy was activated by rapamycin. The fluorescence labeling assay of RFP-GFP-LC3 lentivirus transfection and the electron microscope observation results also confirmed that in BIC-1 and OE33 cells, the incidence of autophagy in the OV group was higher than that in the NC group (Figs. 3C, 4A). Next, we further confirmed the protein interaction between NOD2 and P-ATG16L1 by a co-IP assay (Fig. 4B). Subsequently, ATG16L1-shRNA was used to inhibit the expression of ATG16L1, and autophagy in EA cells was detected by western blotting (Fig. 5). After ATG16L1 was inhibited, the increased NOD2 expression lost its regulatory effect on autophagy in EA cells. We used CCK-8, plate cloning and EDU assays to detect the proliferation of EA cells, and the results (Fig. 4C, D, E) showed that there was no significant difference in proliferation between the OV group and the NC group after ATG16L1 was inhibited. All these results indicate that the NOD2/ATG16L1 pathway is the main pathway through which NOD2 regulates autophagy in EA cells and that upregulation of NOD2 expression inhibits the proliferation of EA cells mainly through the activation of autophagy.

### In vivo experiments confirmed that NOD2 activated autophagy and inhibited proliferation in EA cells

**Fig. 6:**
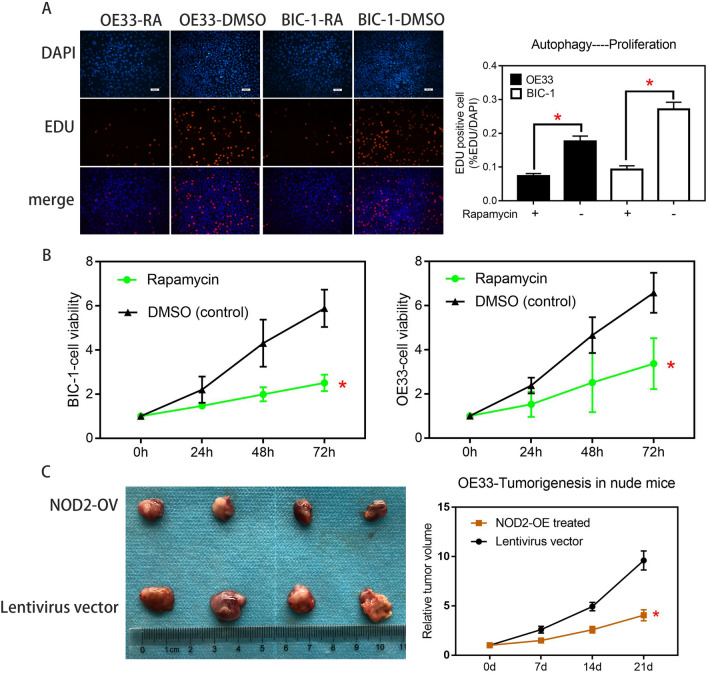
In vivo experiments confirmed that NOD2 activated autophagy and inhibited proliferation in EA cells. The proliferation of OE33 cells and BIC-1 cells before and after rapamycin treatment (100 nM/L) was compared by EDU assays (**A**) and CCK-8 assays (**B**). Statistical results are represented as the mean ± SD (*n* = 3, technical replicates, *P* < 0.05). **C** OE33 cells were implanted in nude mice, and then, we injected the tumors with NOD2-overexpressing lentivirus or lentivirus vector per week for treatment (100 μL, 1*10^7/L) (a total of 3 times). After 21 days, the tumors were removed and photographed. Then, we plotted the tumor growth. Statistical results are represented as the mean ± SD (*n* = 4, biological replicates). *OV group vs. NC group, *P* < 0.05

**Fig. 7:**
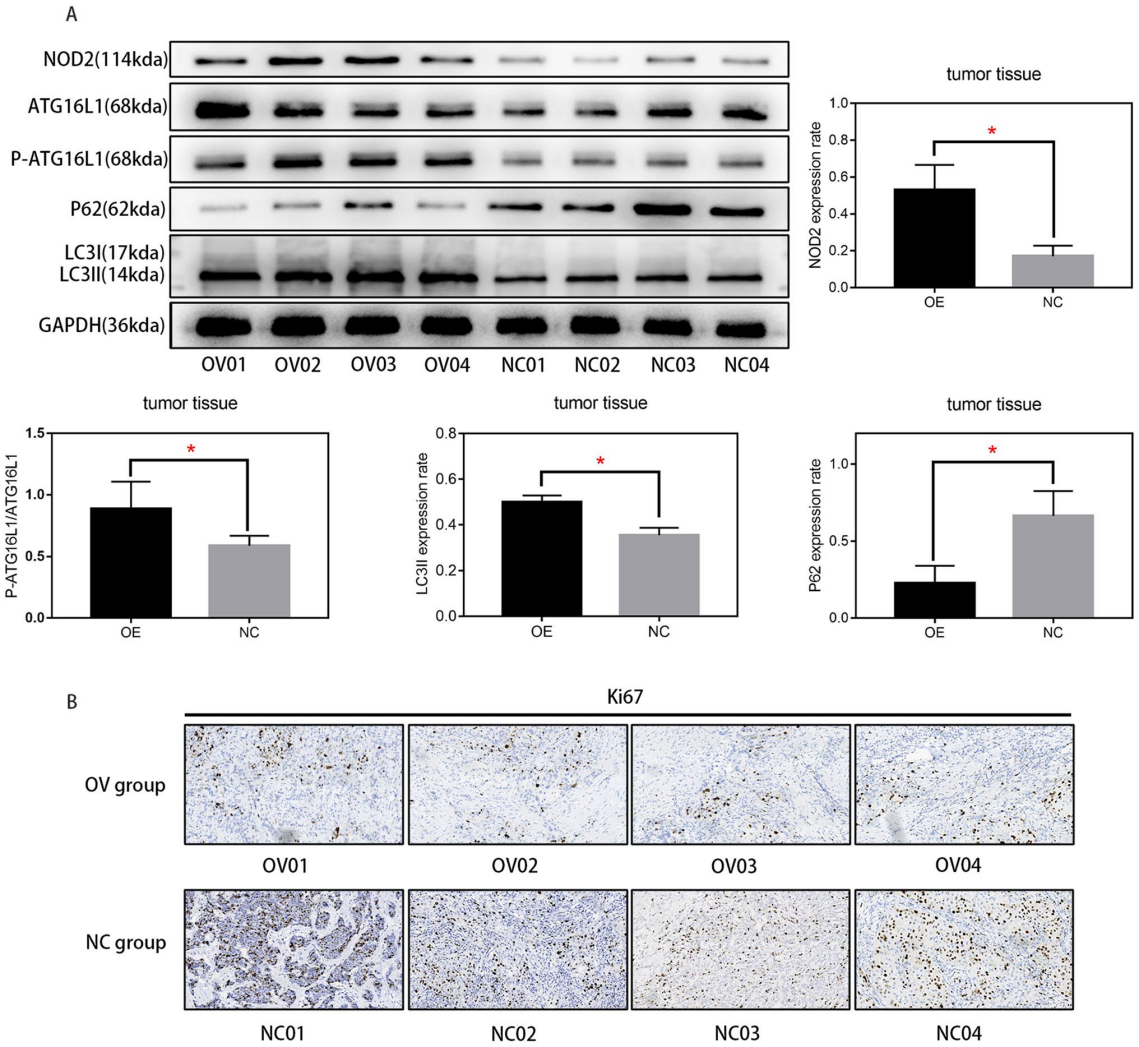
In vivo experiments confirmed that NOD2 activated autophagy and inhibited proliferation in EA cells. **A** Protein was extracted from the tumor tissue shown in Fig. 6C, and the protein expression of ATG16L1, P-ATG16L1, P62, LC3I/II and NOD2 was detected by western blotting. (*n* = 4, biological replicates, *P* < 0.05) **B** The mass of Fig. 6C was sectioned, and Ki67 was labeled with immunohistochemistry and photographed

To verify the effect of autophagy on the proliferation of EA cells, we activated autophagy with rapamycin in BIC-1 and OE33 cells and detected changes in proliferation with EDU and CCK-8 assays. As shown in Fig. 6A and B, in OE33 and BIC-1 cells, the increase in autophagy levels inhibited cell proliferation. Finally, we needed to verify the effect of NOD2 on autophagy and proliferation of EA cells in vivo and determine the practical value of NOD2 in clinical targeted therapy. We conducted a subcutaneous tumorigenesis experiment in nude mice with OE33 cells, injected NOD2 overexpression lentivirus or negative control virus into the tumor tissue every week, measured the size of the tumor, and removed the tumor after three weeks. We measured and weighed the tumor and generated the tumor growth curve. As shown in Fig. 6C, compared with that of the negative control group, the tumor growth rate of the experimental group injected with NOD2 overexpression lentivirus was significantly inhibited. We extracted the protein from the removed mass and detected the protein expression of NOD2, ATG16L1, P-ATG16L1, P62 and LC3I/II by Western blots. The results are shown in Fig. 7A. The injection of NOD2 overexpression lentivirus into the mass successfully increased the expression level of NOD2, increased the protein expression of P-ATG16L1 and LC3II, and reduced the protein content of P62, indicating that the overexpression of NOD2 increased the autophagy level of tumor tissue in vivo. Finally, we sectioned the remaining tumor tissue, labeled the Ki67 target with immunohistochemistry and photographed it. As shown in Fig. 7B, the expression of Ki67 in the OV group was less than that in the NC group, which indicates that the difference in tumor volume is due to the inhibition of tumor cell proliferation in the OV group.

## Discussion

According to the data analysis and experimental verification (Fig. 1A, B, C, D, E, F, H), the expression of NOD2 in EA cells was indeed lower than that in normal cells. Many studies have shown that mutation of NOD2 is closely related to inflammatory bowel disease, intestinal cancer, breast cancer and even endometrial carcinoma (Zhang et al. 2020; Huszno et al. 2020; Hoffmann et al. 2021). The abnormal low expression of NOD2 in esophageal cancer might also be one of the factors that causes the disease (Orlando 2002), but the factors causing the decrease in NOD2 expression are still unclear. Our research group treated EA cells with NOD2 overexpression lentivirus and lentivirus vector and then detected the proliferation of the cells with plate cloning, EDU and CCK-8 assays. The proliferation of the EA cells in the overexpression groups was lower than that of the corresponding control groups (Fig. 2).

First, we need to know how NOD2 regulates esophageal adenocarcinoma cells and how NOD2 inhibits EA cell proliferation. The KEGG analysis (Fig. 3A) showed that in OE33 cells, the upregulation of NOD2 expression mainly affects the “NOD-like receptor signaling pathway”. In fact, the regulation of autophagy by NOD2 through ATG16L1 is considered to be one of the main roles of NOD2 in inflammation (Homer et al. 2012). Although there are different theories about the effect of autophagy on cell proliferation (White et al. 2015; Devenport and Shah 2019; Ferro et al. 2020), artificially enhanced autophagy is considered to inhibit proliferation (Bai et al. 2019; Chen et al. 2020; Mukherjee et al. 2019). We preliminarily suspect that its inhibitory effect on proliferation in EA cells stems from the activation of autophagy (Luo et al. 2019). NOD2 will recruit ATG16L1 protein and combine with ATG5 and ATG12 (Kharaziha and Panaretakis 2017; Wible et al. 2019) to form a complex, which determines the location of autophagy. This pathway has been confirmed in many studies of inflammatory bowel diseases (Homer et al. 2010; Brain et al. 2010). Therefore, we used rapamycin as an autophagy agonist in some experiments to amplify the effect of NOD2 on autophagy (Fig. 3B). We confirmed that overexpression of NOD2 activated the ATG16L1 pathway (Anand et al. 2011) and enhanced autophagy by Western blot detection (Fig. 3B). Through fluorescence (Fig. 3C) and electron microscopy (Fig. 4A), we observed that there were obvious autophagic bodies in the OV group, while the NC group did not have obvious autophagic reactions. Co-IP assays confirmed that there is a strong interaction between NOD2 protein and P-ATG16L1 protein in EA cells, indicating that NOD2 regulates autophagy directly through its interaction with P-ATG16L1. Although the regulatory effect of the NOD2-ATG16L1 pathway on autophagy has been widely studied in IBD (Brain et al. 2010; Travassos et al. 2010; Wang et al. 2014; Hu and Peter 2013), to determine whether the regulation of autophagy by NOD2 mainly comes from ATG16L1, we needed a depletion experiment to determine whether NOD2 participates in autophagic regulation through pathways other than ATG16L1. In fact, there is more than one regulatory mechanism of autophagy by NOD2 in the literature, including common pathways such as NFκB (Wang et al. 2020) and AMPK (Ma et al. 2020), which are regulated by NOD2. However, whether these pathways exist in EA cells and their role in the regulation of autophagy by NOD2 are our next questions. After inhibiting the expression of ATG16L1 in EA cells with ATG16L1-shRNA, we found that NOD2 almost lost the regulation of autophagy and proliferation at almost the same time (Figs. 4C ~ E, 5). This finding indicates that the inhibition of NOD2 overexpression on proliferation in EA cells is mainly due to autophagy mediated by the ATG16L1 pathway.

Another issue is the regulatory relationship between autophagy and proliferation, specifically, the effect of enhanced autophagy on proliferation. Although most studies believe that autophagic activation will inhibit proliferation (Wang et al. 2018; Chen and Gibson 2021; Yu et al. 2020), we still need experimental verification for confirmation. After rapamycin was used to treat OE33 and BIC-1 cells, changes in proliferation were detected by EDU and CCK8 assays (Fig. 6A, B). Not surprisingly, the increase in autophagy inhibited the proliferation of EA cells. After exploring the mechanism by which NOD2 inhibits EA cell proliferation, we still need to consider how practical this mechanism is. Since our previous functional experiments and mechanistic experiments are cell-based in vitro experiments, we needed in vivo experiments to confirm that this anticancer effect can still work in a complex human in vivo environment. As shown in Figs. 6C, 7A, B, the results of the in vivo test are consistent with those of the in vitro test.

Based on the above experimental results and literature analysis, we confirmed that the upregulation of NOD2 expression inhibited the proliferation of EA cells, which was based on the enhanced autophagy due to the interaction between NOD2 and ATG16L1, which led to a decrease in EA cell proliferation. In vivo, the inhibition of NOD2 overexpression lentivirus on the growth of OE33 cells confirmed the feasibility of this mechanism as a new target for gene therapy of esophageal adenocarcinoma. In addition, the anticancer mechanism involved in this study positively regulates the existing biochemical reactions of the human body. Compared with the popular targeted drugs that rely on several gene site mutations, gene therapy has a wider scope of application and has no limitations for the applicable population. However, this study also has shortcomings. Because we did not use normal esophageal epithelial cells as a control, we do not know whether this treatment has side effects on patients’ normal tissues. Due to the limitations of gene therapy itself, it cannot be used as the main treatment for cancer, but I believe it has the potential to become an important adjuvant treatment, inhibit the growth of cancer tissue and provide more time for the preparation of surgery or radiotherapy and chemotherapy. In a follow-up study, we will explore the influencing factors of this mechanism in more cell lines and tissues and will also study the role of this mechanism in normal esophageal cells and conduct control experiments. We plan to further explore the causes of this mechanism and verify it in more cell lines, clinical samples and animal experiments.

## Conclusion

NOD2 can activate autophagy in esophageal adenocarcinoma cells through the ATG16L1 pathway and inhibit cell proliferation.

## Data Availability

The datasets generated during and/or analyzed during the current study are available from the corresponding author on reasonable request.

## Abbreviations

EA: Esophageal adenocarcinoma
NOD2: Nucleotide binding oligomerization domain containing 2
RA: Rapamycin
OV: NOD2 overexpression
NC: Lentivirus vector treatment/negative control
DMSO: Dimethyl sulfoxide vector
